# Relationship Between the Size–Frequency Distribution of Nucleopolyhedrovirus Occlusion Bodies and Their Insecticidal Characteristics on *Spodoptera frugiperda* (Lepidoptera: Noctuidae)

**DOI:** 10.3390/v18050570

**Published:** 2026-05-19

**Authors:** Cristian Ángel-García, Rodrigo Lasa, Joel E. López-Meza, Selene Ramos-Ortiz, Trevor Williams, Ana Mabel Martínez-Castillo

**Affiliations:** 1 Instituto de Investigaciones Agropecuarias y Forestales, Universidad Michoacana de San Nicolás de Hidalgo, Tarímbaro 58880, Michoacán, Mexico; 2026561g@umich.mx; 2 Instituto de Ecología AC, Xalapa 91073, Veracruz, Mexico; rodrigo.lasa@inecol.mx; 3 Centro Multidisciplinario de Estudios en Biotecnología, Facultad de Medicina Veterinaria y Zootecnia, Universidad Michoacana de San Nicolás de Hidalgo, Tarímbaro 58880, Michoacán, Mexico; elmeza@umich.mx; 4 Secretaría de Ciencia, Humanidades, Tecnología e Innovación (SECIHTI), Instituto de Investigaciones Agropecuarias y Forestales, Universidad Michoacana de San Nicolás de Hidalgo, Tarímbaro 58880, Michoacán, Mexico; selene.ramos@umich.mx

**Keywords:** *Baculoviridae*, fall armyworm, SfMNPV, OB cross-sectional area, glycerol density gradient, insecticidal properties

## Abstract

The Spodoptera frugiperda multiple nucleopolyhedrovirus (SfMNPV) is an important pathogen of the fall armyworm and is used as the basis for biological insecticides. In this study, we examined the relationship between the size–frequency distribution of SfMNPV occlusion bodies (OBs) and their insecticidal characteristics when collected at the end of the replication cycle. Exposure of OBs to 40%, 70%, and 90% (*wt*/*wt*) glycerol had no effect on OB pathogenicity. Glycerol density gradient (50–100%) centrifugation was used to separate OBs into two fractions. OBs recovered from the upper fraction of the gradient had a significantly smaller median cross-sectional area than those harvested from the lower fraction. These fractions also differed significantly in their size–frequency distributions. The OB concentration–mortality response of *S. frugiperda* second instars did not differ significantly between the two fractions or with non-centrifuged OBs. The median survival time was similar for insects inoculated with OBs from the upper and lower fractions but was significantly shorter in insects inoculated with non-centrifuged OBs. The proportion of mature OBs (67–71%) and the number of viral genome copies (1.33–1.40 × 10^8^ copies/µL) did not differ significantly between the upper and lower OB fractions. These findings suggest that altering the size–frequency distribution by density gradient centrifugation is not a useful technique for selecting large OBs with high insecticidal activity as part of the baculovirus insecticide production process. Future studies should evaluate a range of OB size separation techniques to determine their effects on OB insecticidal characteristics.

## 1. Introduction

Lepidopteran nucleopolyhedroviruses (genus *Alphabaculovirus*, family *Baculoviridae*) have been widely studied due to their virulence and host specificity against caterpillar pests, which makes them excellent candidates for the development of microbial insecticides [1]. They also form the basis for highly efficient protein expression systems with a range of medical and biotechnological applications [2]. These viruses produce two structurally distinct forms of virions, occlusion-derived virions (ODV) and budded virions, which have separate functions in insect-to-insect and cell-to-cell transmission, respectively. The ODVs comprise nucleocapsids that are enveloped individually or in groups and are occluded in a crystalline protein occlusion body (OB) [3]. The OB protects the ODVs in the environment, both on plant surfaces and in the soil [4,5].

The OB matrix of nucleopolyhedroviruses is mainly composed of polyhedrin in association with fibrillar structures of P10 protein [6,7]. Each OB is covered by outer layers of polyhedron envelope protein (PEP) that provide the mature OB with a smooth, sealed exterior surface [8]. In contrast, immature OBs or those lacking PEP or P10 have deeply pitted surfaces [3].

The Spodoptera frugiperda multiple nucleopolyhedrovirus (SfMNPV) has been isolated from the fall armyworm, *Spodoptera frugiperda* (Lepidoptera: Noctuidae), in its native region of the Americas and also in countries in which it is an invasive pest [9,10,11]. Intensive efforts are currently underway to achieve effective control of this pest, including the development and testing of biological insecticides [12]. SfMNPV can cause natural epizootics in fall armyworm populations [13], which has motivated studies focused on the efficacy of SfMNPV-based biological insecticides [9,14,15,16,17].

Studies on a Nicaraguan isolate of SfMNPV reported high heterogeneity in OB size, with the cross-sectional area of OBs ranging from 0.11 to 6.23 μm^2^ [18]. These authors reported that larvae that died 3–4 days after inoculation produced smaller OBs and a higher proportion of immature OBs compared to larvae that died at 6–7 days post-inoculation. This difference was associated with an increase in pathogenicity for OBs collected from larvae that died at later time points. In a different study, Spodoptera litura nucleopolyhedrovirus (SpltNPV) OBs collected from virus-killed larvae were larger and more pathogenic than OBs collected from infected insects prior to death [19].

Based on these findings, we examined whether the relationship between OB size–frequency distribution and insecticidal activity is an important factor to consider when selecting a baculovirus strain for use in biological pest control. To address this question, we used a glycerol density gradient to separate OBs of different sizes and investigated the pathogenicity and speed of kill of OBs from the resulting fractions in *S. frugiperda* larvae.

## 2. Materials and Methods

### 2.1. Insect Rearing

*Spodoptera frugiperda* larvae were obtained from a colony maintained in the Instituto de Ecología AC (INECOL), Xalapa, Veracruz, Mexico. The colony was known to be free of inapparent SfMNPV infection [20]. The larvae were fed on a semi-synthetic diet modified from Mihm [21]. Newly emerged adults were placed in brown paper bags for oviposition and were fed with a 10% honey solution. The colony was maintained at 26 ± 1 °C, 70 ± 10% relative humidity (RH), and a photoperiod of 14 h:8 h (L:D).

### 2.2. Virus Amplification and OBs Purification

A characterized, genotypically diverse Nicaraguan SfMNPV isolate (Sf-NIC) was used in this study [22]. The virus was amplified in newly molted fourth-instar *S. frugiperda* larvae using the droplet feeding method [23]. To this end, larvae were individualized and starved for 8–12 h and then allowed to drink from an aqueous suspension (1 × 10^8^ OB/mL) containing 10% (*w*/*v*) sucrose and 1% (*v*/*v*) blue food coloring (Bakersfield Blue, Chocolatera de Nayarit, Zapopan, Mexico). Larvae that ingested the OB suspension were individually transferred to the wells of a 24-well tissue culture plate containing semi-synthetic diet. Larvae were incubated at 25 ± 0.5 °C and 65 ± 5% RH in darkness and monitored daily until death. When larvae became moribund and showed clear signs of advanced infection, groups of 10 larvae were transferred to 2 mL centrifuge tubes. After death, larvae were stored at −20 °C until further use.

For OB extraction, virus-killed larvae were thawed and triturated in 0.01% (*w*/*v*) sodium dodecyl sulfate (SDS) solution using a glass tissue homogenizer. The OB suspension was filtered through an 80 μm metal mesh to remove tissue debris. A 9 mL volume of the OB suspension was placed on 9 mL of 40% (*w*/*w)* glycerol and centrifuged at 9300× *g* for 30 min. The resulting pellet was washed with 15 mL of sterile ultrapure water and centrifuged at 5900× *g* for 15 min, after which it was resuspended in 5 mL of sterile ultrapure water. The procedure was performed ten times and the resulting OB suspensions were pooled to obtain a final volume of 50 mL. The OB concentration was determined by counting triplicate samples using a Neubauer hemocytometer (Hawskley, Lancing, UK) under phase contrast microscopy (Olympus BX41; Guadalajara, Jalisco, Mexico) at ×400. The OB suspension was stored at 4 °C until use.

### 2.3. Effect of Glycerol on OB Pathogenicity

The potential effect of different glycerol concentrations on the biological activity of SfMNPV OBs was tested as follows. A 1 mL suspension of 1 × 10^7^ OBs was centrifuged at 9000× *g* for 10 min, after which the supernatant was discarded. The OB pellet was resuspended in 1 g of 40, 70 or 90% (*w*/*w*) glycerol or 1 g of sterile ultrapure water, which served as a virus control. Sterile ultrapure water without OBs was included as a negative control. Ten independent replicates were prepared for each OB + glycerol mixture, as well as for the positive and negative controls.

Each OB–glycerol mixture was incubated for 1 h at room temperature (~20 °C). Sterile ultrapure water was then added to each sample to obtain a final volume of 1.5 mL. The sample was then mixed thoroughly on a vortex mixer and centrifuged at 13,000× *g* for 15 min. The supernatant was discarded and the OB pellet was washed with 1 mL of sterile ultrapure water. The sample was again mixed thoroughly with a vortex mixer and centrifuged at 9000× *g* for 10 min. The resulting pellet was resuspended in 1 mL sterile ultrapure water. OBs were then counted from triplicate samples of a Neubauer counting chamber at ×400 magnification.

The OB suspension of each replicate and treatment was adjusted to a concentration of 5.3 × 10^5^ OBs/mL in sterile ultrapure water containing 10% (*w*/*v*) sucrose and 1% (*v*/*v*) blue food coloring. This concentration was expected to kill 50% of second-instar *S. frugiperda* larvae, based on previous preliminary tests. For the bioassays, groups of 24 larvae were starved overnight as they molted to the second instar and were inoculated with each OB suspension using the droplet feeding method. Larvae that ingested the inoculum within 15 min were individually transferred to the wells of a 24-well tissue culture plate containing a semi-synthetic diet and maintained in a bioclimatic chamber at 25 ± 0.5 °C, 65 ± 5% RH, in darkness. Control larvae ingested only sucrose solution and food coloring without OBs. Mortality was recorded daily up to 12 days post-inoculation.

### 2.4. Separation of OB Samples

As the previous experiment indicated no adverse effects of glycerol exposure, a series of glycerol concentrations of 50, 55, 60, 65, 70, 75, 80, 85, 90 and 100% (*w*/*w*) was prepared using sterile ultrapure water. Discontinuous density gradients were prepared by adding 1 g of each glycerol concentration to a 17 mL conical centrifuge tube to produce approximately 9 mL of gradient per tube. An 8 mL volume of filtered OB suspension (2.4 × 10^9^ OBs/mL), prepared as described in Section 2.2, was carefully placed on top of the density gradient. Centrifugation was performed at 18 °C and 13,400× *g* for 1 h in a refrigerated centrifuge equipped with a 13/004-221.18 fixed-angle 38° rotor (Hermle Labortechnik, Z36-HK, Wehingen, Germany). This procedure was performed nine times (replicates).

In all cases, the OBs formed a distinct vertical streak along the inner wall of the centrifuge tube, located between the 50% glycerol and 90% glycerol layers. The length of each vertical streak of OBs was measured and subdivided into three fractions: the upper 35%, the central 30%, and the lower 35%. The glycerol densities at these positions were 1.096–1.140 g/cm^3^, 1.151–1.174 g/cm^3^ and 1.184–1.254 g/cm^3^ for the upper, central and lower fractions, respectively, when the tube was in a vertical position, although the density gradient will have shifted during centrifugation at a 38° angle. Each fraction was collected by cutting the tubes transversely with a sterile blade and OBs were gently scraped off the wall of the tube using a sterile toothpick. To eliminate glycerol residue, each OB sample was divided among three sterile microcentrifuge tubes with 1.5 mL of sterile ultrapure water, vortexed, and centrifuged at 5600× *g* for 10 min. The supernatant was discarded, and the OB pellet was resuspended in 333 μL of sterile ultrapure water. The three suspensions were then pooled in a single tube to produce 1 mL of OB suspension for each of the upper, central and lower fractions collected from the density gradient. The OB concentration was determined by counting in triplicate using an improved Neubauer hemocytometer.

### 2.5. OB Size and Maturity

Four OB samples (four randomly selected replicates) from the upper, central, or lower fractions harvested from the density gradients (Section 2.4) were used to determine the OB size and maturation. For this, OBs from each replicate were examined in an FEI Quanta 250 FEG scanning electron microscope (SEM) (FEI Company, Hillsboro, OR, USA). A 100 μL volume of OB suspension of each replicate was adjusted to 5 × 10^7^ OBs/mL by the addition of ultrapure water. The four suspensions were pooled and mixed in a vortex and 20 μL droplets were pipetted onto an aluminum stub. The droplets were allowed to dry for 10 h at room temperature and examined without sputter coating at ×10,000 magnification and an accelerating voltage of 5.00 kV. Images obtained were captured, manually edited to a binary format (black and white), and the cross-sectional area of each OB was measured with reference to a 20 μm scale bar using ImageJ 1.54p Java 25.0.1 software [24]. A total of 118 images were analyzed at a resolution of 72 ppi, consisting of 35, 36, and 47 images for the OB samples from the upper, central, and lower fractions of the glycerol gradient samples (Section 2.4), respectively. This resulted in the measurement of 1301, 1559, and 1417 OBs for each fraction, respectively.

The prevalence of mature OBs was only determined for OB-enriched fractions from the upper and lower samples of the glycerol gradient (Section 2.4). As uncoated samples did not provide sufficient resolution for the determination of OB maturity, OBs were coated with gold–palladium in a Q150R sputter-coater (Quorum, Laughton, UK) and were observed at ×10,000 magnification. A total of 38 and 49 images (3944 and 5226 OBs in total) were analyzed from four replicate samples of the upper and lower fractions, respectively. As the outer layers of PEP protein result in a smooth OB surface, OBs were classified as mature if they had a smooth, continuous surface and immature if they showed the presence of pits or cavities on the OB surface [8].

As the results of this experiment and the study on the biological activity of OBs (Section 2.6) raised questions on the viability of OBs that had been subjected to density gradient centrifugation, three additional replicate batches of SfMNPV OBs were amplified as described in Section 2.2. These batches were subjected to OB extraction in ultrapure water and subjected to one of three treatments: (i) filtration through 80 µm steel mesh without centrifugation, (ii) filtration followed by 40% glycerol cushion centrifugation (Section 2.2), or (iii) filtration and incubation in ultrapure water or 0.01% SDS for 3 h at 23 °C to determine the effect of SDS treatment on OB integrity. Samples from each of these treatments were examined in the scanning electron microscope at ×5000 and photographed at the same resolution as described for the density gradient samples. For analysis of the prevalence of mature and immature (pitted) OBs, a total of 15 images (filtration alone), 30 images (filtration + 40% glycerol), and 8–11 images (ultrapure water and 0.01% SDS) were analyzed, from which a total of 330, 1274, and 868–915 OBs were examined, respectively. For OB area measurements from the filtered samples (no centrifugation), a total of 39 images and 952 OBs were analyzed.

### 2.6. Insecticidal Properties of OBs

The OB concentration–mortality response of second-instar *S. frugiperda* larvae was determined using pooled samples from four randomly selected replicates of the upper and lower fractions of density gradients (Section 2.4). An OB suspension without centrifugation in a glycerol gradient was also included as a positive control, and a solution without OBs was the negative control. For the bioassay, first-instar *S. frugiperda* larvae were starved overnight and those that molted to the second instar were inoculated using the droplet-feeding method with one of the following concentrations, 1 × 10^7^, 1 × 10^6^, 3 × 10^5^, 3 × 10^4^, or 3 × 10^3^ OBs/mL, which were expected to result in 10–90% lethal polyhedrosis disease based on preliminary tests. Control larvae were inoculated with a solution of sucrose and food coloring without OBs. Groups of 24 inoculated larvae from each concentration were placed individually in the wells of a 24-well tissue culture plate containing a piece of semi-synthetic diet and incubated at 27 ± 0.5 °C, 70 ± 10% relative humidity in darkness. The bioassay was conducted with eight replicates for the upper and lower OB fractions using different batches of insects and including six replicates for the positive control (non-centrifuged OBs). Mortality was recorded at 8 h intervals over a 9-day period.

### 2.7. DNA Extraction and Quantification of Viral Genomes

Samples of 1 × 10^8^ OBs in 200 μL ultrapure water were obtained for five randomly-selected replicates of the upper and lower OB fractions (Section 2.4). Virions were released from the OBs by incubation with 100 μL of 3× DAS buffer (0.51 M NaCl, 0.3 M Na_2_CO_3_, 30 mM EDTA) for 5 min at 40 °C. Undissolved OBs were pelleted by centrifugation at 1000 rpm for 1 min and discarded. The supernatant containing the virions was mixed with 25 μL of proteinase K (20 mg/mL) and 25 μL of 10% SDS and incubated at 40 °C for 1 h. Viral genomic DNA was then extracted with phenol and twice with phenol–chloroform and precipitated by the addition of 3 M sodium acetate and ice-cold ethanol. The precipitate was centrifuged at 16,700× *g* for 15 min, washed with 96% and 70% ethanol and dissolved in 70 µL of TE buffer (10 mM Tris, 1 mM EDTA, pH 8.0). The DNA concentration of each sample was then measured in a spectrophotometer (BioSpec-Nano, Shimadzu, Kyoto, Japan).

Quantitative PCR (qPCR) based on SYBR fluorescence was performed in an Mx3005P qPCR System (Agilent Stratagene, Santa Clara, CA, USA) in 96-well reaction plates. Primers were designed based on the polyhedrin gene sequence, forward primer (5′-GAACCTTCACTCTGAGTACACGCAC) and reverse primer (5′-AGACGATGGGTTTGTAGAAGTTCTCC), which amplified an 82 bp region of the *polh* gene. The amplification program consisted of 3 min at 95 °C, followed by 40 cycles of 30 s at 95 °C, 30 s at 60 °C and 1 min at 72 °C. Each sample was amplified in triplicate reactions as described by Molina-Ruiz et al. [20]. Ultrapure water was used as a negative control.

### 2.8. Statistical Analysis

Data were tested for homoscedasticity and normality by Levene’s and Shapiro–Wilk tests, respectively, prior to analysis. The percentage of mortality of *S. frugiperda* larvae that consumed OBs treated with or without glycerol was adjusted using Abbott’s [25] correction for control mortality (2.6%) and subjected to one-way analysis of variance (ANOVA). Larval survival from tests on glycerol (40, 70 or 90%) and OB concentration–mortality bioassays were subjected to Kaplan–Meier survival analysis and compared by log-rank test. OB cross-sectional area values were not normally distributed and were subjected to Kruskal–Wallis non-parametric analysis. Medians were compared by Dwass–Steel–Critchlow–Fligner (DSCF) pairwise comparisons. The distribution of OB cross-sectional area values from upper and lower fractions was adjusted to a normalized probability distribution with identical bin edges and overlapping kernel density estimates (KDE) [26]. A two-sample Kolmogorov–Smirnov test was used to compare the frequency–size distributions of OBs from the upper and lower fractions. The proportions of immature OBs and viral DNA copies (qPCR) were compared by *t*-test. These analyses were performed using the R-based software Jamovi v.2.4.66 [27]. The OB concentration–mortality response was subjected to logit regression using the Generalized Linear Interactive Modelling (GLIM 4) software [28] with a quasibinomial error structure to account for minor overdispersion.

## 3. Results

### 3.1. Effect of Glycerol on OB Pathogenicity

**Figure 1:**
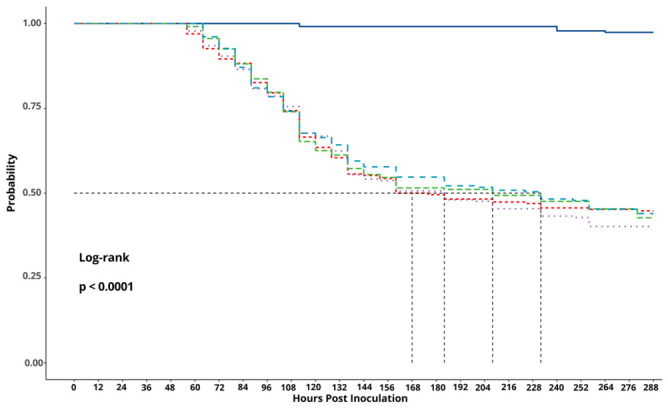
Kaplan–Meier survival curves comparing the pathogenicity of the SfMNPV-NIC isolate OBs in *S. frugiperda* larvae following exposure to glycerol gradients at 40% (blue dashed line), 70% (green dashed line), 90% (purple dotted line), OBs in water (positive control, red dashed line) and only water alone (negative control, dark blue solid line). The horizontal dashed line indicates the 50% survival probability threshold, while the vertical dashed lines indicate the median survival time for each treatment.

No significant differences were observed in the mean (±SE) larval mortality of second-instar larvae that consumed OBs that had been exposed to 40, 70, and 90% glycerol, which resulted in 56.6 ± 2.0, 54.9 ± 2.2, and 59.2 ± 1.8% mortality, respectively, compared to 53.3 ± 2.4% mortality in the positive control (*F* = 1.42; df = 3, 36; *p* = 0.25). Similarly, no significant differences were observed in the median survival time of second-instar larvae that consumed OBs that had been exposed to glycerol (ranging from 184 to 232 h post-inoculation) compared to the positive control (168 h post-inoculation, *p* > 0.05), but these all differed significantly from the negative control (97–99% survival) (log-rank test, *χ*^2^ = 184, df = 4, *p* < 0.001, Figure 1).

### 3.2. OB Size and Maturity

SfMNPV OBs varied in size across all fractions analyzed, with cross-sectional area ranging from 0.46 to 6.6 μm^2^. The OBs obtained from the upper fraction had a significantly smaller cross-sectional area than OBs from both the central and the lower fractions, which did not differ from one another (Kruskal–Wallis, *χ*^2^ = 760.5, df = 3, *p* < 0.001; Table 1). Based on this result, the OB samples from the central fraction were excluded from subsequent studies to focus on the difference between the upper and lower fractions alone. In contrast, despite being amplified under the same conditions, non-centrifuged samples amplified separately from the density gradient samples were significantly smaller in terms of the minimum, maximum and median cross-sectional area.

**Figure 2:**
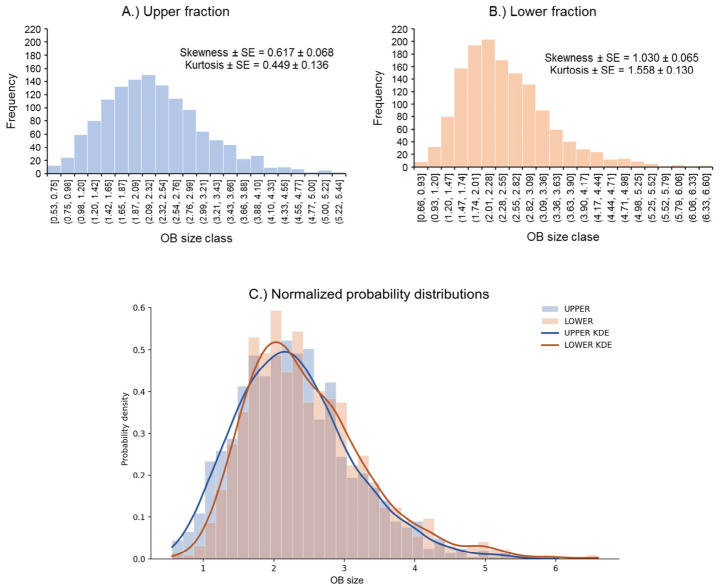
Comparison of frequency–size distributions of OBs from (**A**) the upper fraction (blue) and (**B**) the lower fraction (peach) of glycerol centrifugation gradients and (**C**) normalized probability distributions of both fractions with identical bin edges and overlaid kernel density estimates (KDE).

A higher degree of skewness and kurtosis was observed in the size–frequency distributions of OBs from the lower fraction than those from the upper fraction (Figure 2A,B), i.e., the lower fraction comprised a more strongly right-skewed distribution (larger OBs) with a sharper central peak (leptokurtic) and extreme values in the right tail (the largest OBs). The OB size–frequency distributions differed significantly between fractions (Kolmogorov–Smirnov, *D* = 0.0793, *p* < 0.0004), although these differences were not marked, and when normalized to facilitate direct comparison, the probability distributions showed a high overlap coefficient (0.93; Figure 2C).

**Figure 3:**
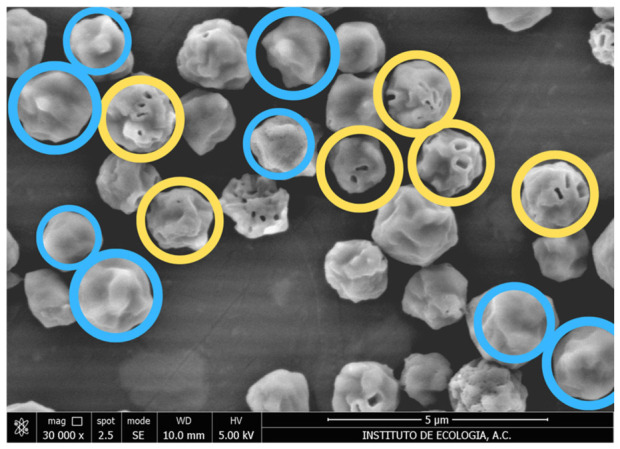
Scanning electron photomicrograph of mature (blue circles) and immature OBs (yellow circles) observed at ×30,000. Mature OBs have a smooth, sealed exterior surface, whereas immature OBs show the presence of cavities and irregular surfaces. OBs whose maturity status could not be clearly determined were not counted.

No significant difference was observed between the mean (±SE) percentage of mature OBs from the upper (67.4 ± 3.3%) and lower fraction (70.8 ± 2.2%) (*t* = −0.715; df = 6; *p* = 0.502) (Figure 3). To ensure that the prevalence of immature OBs with a pitted appearance was not an artefact of exposure to SDS or centrifugation steps, additional studies were performed. The appearance of OBs that had been exposed to 0.01% SDS for 3 h at 23 °C was identical to that of OBs incubated in ultrapure water for the same period, indicating that this concentration of SDS did not influence the prevalence of OBs with pitted surfaces (Supplemental Figure S1). Similarly, the prevalence of pitted OBs in OB suspension that was extracted from virus-killed larvae and filtered through 80 µm steel mesh did not differ significantly from OB samples that were subjected to centrifugation through a 40% (*w*/*w*) glycerol cushion at 9300× g for 30 min (Section 2.2), although both of these treatments resulted in a slightly higher mean (±SE) prevalence of pitted OBs (37.0 ± 2.8–37.1 ± 1.6%, respectively), compared to OB samples from the upper and lower fractions of the glycerol density gradients (mean prevalence 32.8 and 32.1%, respectively) (*F* = 2.852, df = 3, 128; *p* = 0.040) (Supplemental Figure S2). This finding was consistent with the pitted appearance of OBs, reflecting their earlier stage of development rather than artefacts induced by low concentration SDS use or centrifugation steps during the experimental procedures.

### 3.3. Concentration–Mortality Bioassays and Quantification of Viral Genomes

No significant differences were detected in the concentration–mortality response of insects inoculated with OBs from the upper and lower fractions, or when these were compared to the non-centrifuged samples (Table 2).

**Figure 4:**
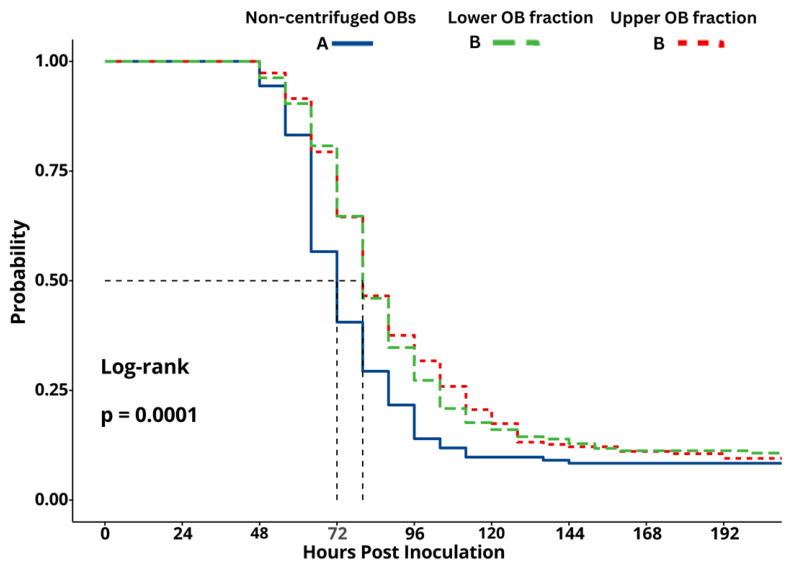
Kaplan–Meier survival curves of second-instar *Spodoptera frugiperda* larvae inoculated with a concentration of 1 × 10^7^ OBs/mL of SfMNPV. Fractions comprised OBs from the upper fraction (red line) and lower fraction (green line) obtained by glycerol density gradient centrifugation, as well as non-centrifuged OBs (blue line). Vertical dashed lines indicate median survival times. Survival curves followed by identical upper case letters did not differ significantly (log-rank test, *p* > 0.05).

Kaplan–Meier survival analysis detected a significant difference in survival between larvae inoculated with non-centrifuged samples and those inoculated with OBs from the upper and lower fractions (log-rank test, *χ*^2^ = 19.9, df = 2, *p* < 0.001, Figure 4). The median survival time of larvae inoculated with the non-centrifuged OBs was estimated at 72 h post-inoculation compared to 80 h post-inoculation for the upper and lower OB fractions (Figure 4).

No significant differences were observed in the mean (±SE) genome copy number between the upper (1.33 × 10^8^ ± 1.19 × 10^7^ copies/µL) and lower OB fractions (1.40 × 10^8^ ± 1.01 × 10^7^ copies/µL) (*t* = 0.503; df = 16; *p* = 0.62).

## 4. Discussion

The size of nucleopolyhedrovirus OBs and their insecticidal characteristics vary at different stages of the infection cycle [18,19]. In this study, we determined the phenotype of SfMNPV OB fractions obtained by glycerol density gradient centrifugation. A high heterogeneity in SfMNPV OB size was observed (0.46–6.6 µm^2^) when these were collected from upper and lower fractions or in a smaller range (0.36–4.38 µm^2^) for the non-centrifuged samples (Table 1). A wide range of sizes has been observed in OBs of both SfMNPV (0.11–6.23 μm^2^ area) [18] and BmNPV (2.2–6.5 μm diameter) [8] harvested from dead larvae and an insect cell culture, respectively. This variability could be related to the development and maturation of the OBs during the late stages of infection [18]. Alternatively, Sajjan and Hinchigeri [8] postulated that the high heterogeneity of OB size could be related to resource and space constraints within the virogenic stroma of the host nucleus. Unexpectedly, the non-centrifuged OBs that were amplified separately to examine the potential effects of SDS treatment and centrifugation-induced physical stress were significantly smaller than the batches produced for density gradient centrifugation, despite being generated using the same insect colony under identical laboratory conditions. We can only speculate that this difference resulted from subtle variation arising during viral replication within the host, possibly due to host stress or physiological variation of unknown etiology. For this reason, we did not pursue a detailed comparison of the OB size distributions between density gradient and non-centrifuged OB preparations.

In our study, the frequency–size distribution of OBs differed across fractions. The lower OB fraction comprised an OB population with a 5% higher mean cross-sectional area than OBs from the upper fraction. Assuming that OBs approximate spheres, this difference in area would equate to a 7.6% difference in OB volume between the upper and lower fractions. The original idea of separating OBs by centrifugation into fractions that differed in OB size was based on the notion that small and large OBs would differ in density and sedimentation rate. For example, an early study demonstrated that the buoyant density of OpMNPV OBs ranged from approximately 1.255 to 1.265 g/cm^3^ with two peaks at 1.260 and 1.264 g/cm^3^. The OBs from these peaks differed in virus content, with the larger (denser) OBs having 29% more nucleocapsids per OB and 28% more nucleocapsids per ODV compared to the smaller OBs [29]. Estimates of the buoyant density of other nucleopolyhedrovirus OBs vary from 1.193 to 1.268 g/cm^3^ [30,31]. Consequently, the range of buoyant densities within each sample likely reflects the variation in both OB size and ODV and nucleocapsid content of the sample.

For ODVs, the buoyant density is close to that of OBs, with estimates of 1.15–1.24 g/cm^3^ in SfMNPV [32] and estimates in the range of 1.20–1.30 g/cm^3^ for other multinucleocapsid nucleopolyhedroviruses [29,31,33]. The buoyant density of ODVs also increases according to the nucleocapsid content in AcMNPV [33]. It was therefore reasonable to assume that centrifugation through a density gradient would result in the sorting of OBs according to their size and ODV/nucleocapsid content. Although the size–frequency distributions of the upper and lower fractions differed significantly, there was a high degree of overlap and only a small difference in median OB size.

Given that we collected OBs from the side of centrifuge tubes, it is unclear whether the OB samples had reached their isopycnic point at which the particle’s buoyant density matches the density of the surrounding medium. The use of a fixed-angle rotor rather than a swing-out rotor allows for faster separations and improved resolution, although particles may strike the tube wall before reaching their isopycnic position. In our samples, the fixed-angle rotor would have shifted the position of the gradient, which resulted in the deposition of OBs on the tube wall from where they could easily be classified into upper and lower fractions and recovered for processing. However, the use of a swing-out rotor and the collection of OBs suspended in glycerol layers could have resulted in a different OB size–frequency distribution, although this equipment was not available to us and was not tested. This represents an important limitation of the present study. Future investigations should employ more robust particle-separation methodologies, such as isopycnic density gradient centrifugation or flow cytometric sorting, to achieve more precise fractionation of OB populations according to size.

In this study, no significant variation in OB pathogenicity was observed among the different OB fractions. In contrast, previous studies on SfMNPV [18] and SpltNPV [19] revealed that OBs collected at later stages of infection were associated with increases in both OB size and pathogenicity, which, in the case of SfMNPV, was attributed to a higher prevalence of mature OBs in late infection samples [18]. Unexpectedly, insects inoculated with OBs of non-centrifuged samples had shorter median survival time compared to insects treated with OBs from the upper and lower fractions. Although we initially suspected that the higher virulence of non-centrifuged OBs was due to a lower prevalence of immature OBs in the sample, subsequent studies did not support this idea, as non-centrifuged samples had a marginally higher immature OB composition (mean 36.8%) compared to density gradient samples (32.1–32.8%) (Figure S2). This finding leaves us with two possible explanations: either the stresses experienced during centrifugation resulted in internal damage to the OB or ODVs that was not visible in scanning electron microscope images, or the smaller OBs in the non-centrifuged sample are inherently more virulent than the larger OBs from the density gradient samples (Table 1). This latter idea does not find support from the LC_50_ bioassay (Table 2) or the previous studies of Velasco et al. [18], and would challenge our original hypothesis that larger OBs have higher insecticidal properties than their smaller counterparts. However, if small OBs dissolve and release ODVs more quickly, soon after reaching the alkaline conditions of the midgut, then the ODVs from small OBs may be able to diffuse, cross the peritrophic matrix and initiate infection of midgut cells faster than ODVs from the slower-dissolving large OBs consumed at the same time. This would give the small OB inoculum a temporal infection advantage. Although speculative, this is an intriguing idea that merits further investigation.

In our study, no significant differences were observed in the proportion of mature OBs in the upper and lower fractions (67–71%) or in the number of viral genome copies between these OB fractions (1.33 × 10^8^ vs. 1.40 × 10^8^ copies/µL) in samples collected at 12–24 h after the host’s death. The OB maturation process involving polyhedrin, P10 and layers of PEP protein is critical for virus persistence and transmission in the environment [3,8,34]. In addition, the incorporation of alkaline proteases into OBs during maturation contributes to the disruption of the OB matrix and facilitates ODV release in the host midgut [3,35].

The absence of differences in the proportion of mature OBs and the number of viral genome copies between OB fractions could explain the similarity in OB pathogenicity (LC_50_ values) from upper and lower fractions, regardless of their size. Consistent with our findings, Velasco et al. [18] also reported no differences in viral genome copy number among SfMNPV OBs taken at different moments post-infection. Using transmission electron microscopy, Allaway [36] observed that an isolate of AgseMNPV with large OBs contained more virions and nucleocapsids per OB than smaller OBs from a different isolate of the same virus. An alternative approach would be to directly count the ODVs present within OBs that have been dissolved by alkali treatment and negatively stained for transmission electron microscopy. This technique has been successfully employed in the study of the roles of viral proteins in ODV and OB morphogenesis in AcMNPV and HearNPV [37,38]. This could prove to be a useful approach for future studies comparing OB size and ODV content.

Additionally, the formation and packaging of ODVs and nucleocapsids may be genetically determined in each nucleopolyhedrovirus strain, which would be a confounding factor in the comparison of different strains or isolates [38,39,40,41]. In the present study, DNA extraction and purification involved multiple steps to obtain high-quality DNA with a minimal presence of polymerase inhibitors, which likely resulted in a loss of material and may have masked differences in genome content among our samples.

## 5. Conclusions

We conclude that small differences in the size–frequency distribution of the upper and lower fractions of SfMNPV OBs were not correlated with their insecticidal activity. Although statistically significant separations between size proportions with distinct distributions were achieved, these differences were not important enough to be biologically significant in terms of pathogenicity, the proportion of mature OBs, or viral genome copies. Overall, our findings suggest that, in its current form, size-based separation using a glycerol gradient is unlikely to be a viable strategy for selecting OBs with enhanced insecticidal properties. Although optimizing this novel approach could facilitate the size separation at different stages of the infectious cycle, further studies are needed to understand its possible practical implications.

## Figures and Tables

**Table 1:** Cross-sectional area of SfMNPV OBs (μm^2^) obtained from three fractions collected following glycerol density gradient centrifugation.

Fraction	*N*	Median Area (μm^2^)	25 Percentile	75 Percentile	Minimum Area (μm^2^)	Maximum Area (μm^2^)
Upper	1301	2.21 b	1.71	2.79	0.53	5.44
Central	1559	2.32 c	1.83	2.88	0.46	6.05
Lower	1417	2.32 c	1.85	2.94	0.66	6.60
Non-centrifuged	952	1.54 a	1.19	1.99	0.36	4.38

Non-centrifuged samples were amplified separately from the density gradient samples and were filtered through an 80 µm steel mesh but not centrifuged. *N* indicates the total number of OBs measured in pooled samples examined by SEM. Median values followed by identical letters did not differ significantly (Kruskal–Wallis, DSCF pairwise comparisons, *p* > 0.05).

**Table 2:** Logit regression analysis of the concentration–mortality response and median lethal concentration (LC_50_) of second-instar *Spodoptera frugiperda* larvae inoculated with OBs from the upper and lower fractions following glycerol density gradient centrifugation and non-centrifuged OBs.

OB Fraction	Slope ± SE	Intercept ± SE	LC_50_ (OBs/mL × 10^5^) (95% CI)
Upper	0.548 ± 0.04	−6.961 ± 0.46	3.3 (2.2–4.9) a
Lower	0.440 ± 0.03	−5.661 ± 0.40	3.8 (2.2–6.7) a
Non-centrifuged OBs	0.539 ± 0.04	−7.154 ± 0.55	5.7 (3.6–9.4) a

The error distribution of logit models was scaled to account for moderate overdispersion. Values followed by identical letters did not differ significantly (*t*-test, *p* > 0.05).

## Data Availability

The original contributions presented in this study are included in the article and Supplementary Material File S1. Further inquiries can be directed to the corresponding authors.

## Supplementary Materials

The following supporting information can be downloaded at: https://www.mdpi.com/article/10.3390/v18050570/s1, File S1: File_S1.xlsx Original data; Figure S1. Scanning electron photomicrographs of SfMNPV OBs to examine the effects of SDS treatment; Figure S2. Scanning electron photomicrographs of SfMNPV OBs to estimate the prevalence of immature and mature OBs.

## Abbreviations

The following virus common name abbreviations are used in this manuscript:

AcMNPV	Autographa californica multiple nucleopolyhedrovirus
AgseMNPV	Agrotis segetum multiple nucleopolyhedrovirus
BmNPV	Bombyx mori nucleopolyhedrovirus
HearNPV	Helicoverpa armigera nucleopolyhedrovirus
OpMNPV	Orgyia pseudotsugata multiple nucleopolyhedrovirus
SfMNPV	Spodoptera frugiperda multiple nucleopolyhedrovirus
SpltNPV	Spodoptera litura nucleopolyhedrovirus
