# Supplementary material for: Relationship Between the Size–Frequency Distribution of Nucleopolyhedrovirus Occlusion Bodies and Their Insecticidal Characteristics on Spodoptera frugiperda (Lepidoptera: Noctuidae)
Source: Viruses. 2026 May 19;18(5):570. doi: 10.3390/v18050570 (PMC13211362; doi:10.3390/v18050570)
Supplement: Supplementary file 1 [file viruses-18-00570-s001.zip › Figure S1.pdf]

A) Control **B)** 0.01% SDS

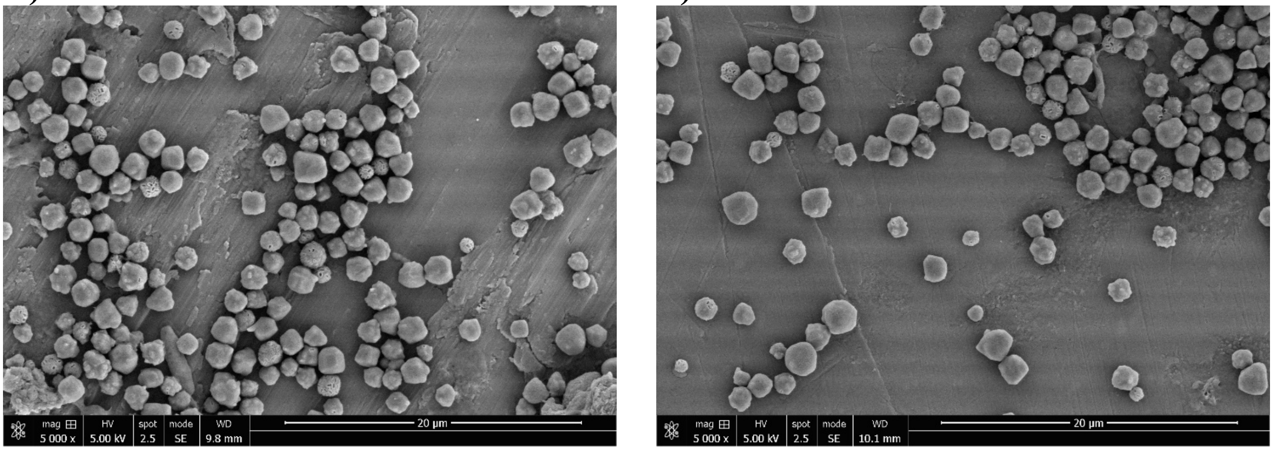

Figure S1. Scanning electron photomicrographs of SfMNPV OBs to examine the effects of SDS treatment. Samples were from (A) control samples and (B) samples treated with 0.01% SDS for 3 h at 23 °C. Samples comprised 100 µL of 1×10^8 OB/mL and were washed once in ultrapure water before being adjusted to 5×10^7 OB/mL, dried on aluminium stubs, sputter-coated and observed at ×5000 in a FEI Quanta 250 FEG scanning electron microscope at 5 kV. The mean (± SE) percentage of immature OBs (with surface pitting) was 28.9 ± 1.8% for the control (8 photomicrographs comprising 868 OBs) compared to 25.6 ± 2.0 for the 0.01% SDS treatment (11 photomicrographs comprising 915 OBs), which did not differ significantly ($t = 1.18$, df = 17, $p = 0.256$). Percentage values were normally distributed (Shapiro-Wilk test) and had equal variances (Levene's test).
