# Supplementary material for: Relationship Between the Size–Frequency Distribution of Nucleopolyhedrovirus Occlusion Bodies and Their Insecticidal Characteristics on Spodoptera frugiperda (Lepidoptera: Noctuidae)
Source: Viruses. 2026 May 19;18(5):570. doi: 10.3390/v18050570 (PMC13211362; doi:10.3390/v18050570)
Supplement: Supplementary file 1 [file viruses-18-00570-s001.zip › Figure S2.pdf]

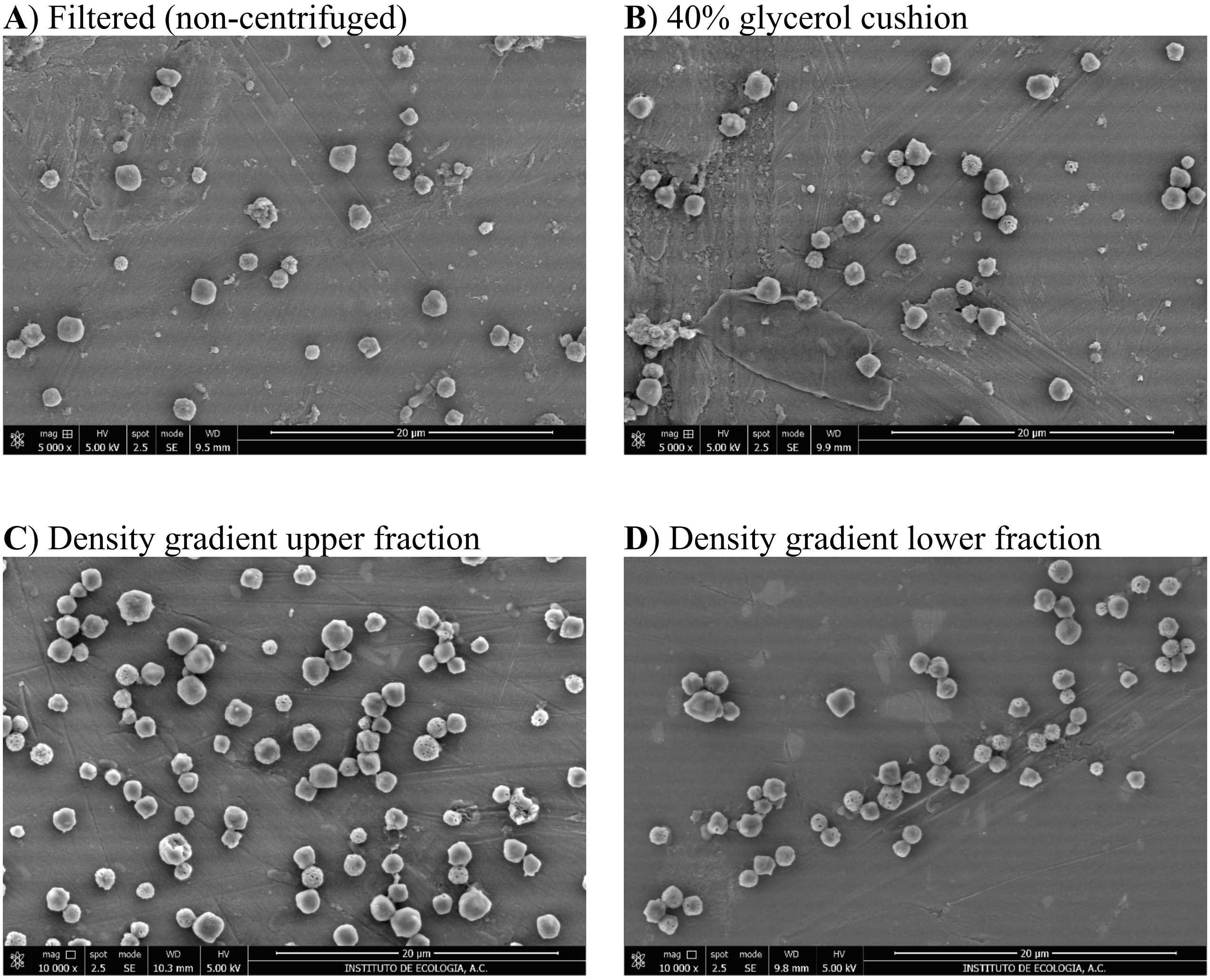


**Figure S2**. Scanning electron photomicrographs of SfMNPV OBs to estimate the prevalence of immature and mature OBs. Samples were from (A) OB suspension extracted from larval cadavers using ultrapure water and filtered through 80 µm steel mesh, (B) OB suspension filtered through steel mesh and passed through a 40% glycerol cushion (9,300×g for 30 min), (C) OBs recovered from the upper fraction of the glycerol density gradient, (D) OBs recovered from the lower fraction of the glycerol density gradient. The mean (± SE) percentage of immature (pitted) OBs in each of the samples was 37.0 ± 2.8% in (A), 37.1 ± 1.6% in (B), 32.8 ± 1.4% in (C), and 32.1 ± 1.2% in (D), which differed marginally ($F = 2.852$; df = 3, 128; $p = 0.040$), but no significant differences were detected between treatment means when compared by Tukey test ($p > 0.05$), likely due to the lower power of the post-hoc test. These percentages were based on the observation of 330, 1274, 3944, 5431 OBs for A-D, respectively. Percentage values were normally distributed (Shapiro-Wilk test) and had equal variances (Levene's test).

The finding that the prevalence of immature (pitted) OBs was marginally higher in the samples that were filtered (A) or subjected to a 40% glycerol cushion (B) indicates that the pitted appearance of a fraction of the OBs was not an artefact of the centrifugation procedures of the use of high concentrations (50 – 100%) of glycerol.
